# Essential Oils as In Vitro Ruminal Fermentation Manipulators to Mitigate Methane Emission by Beef Cattle Grazing Tropical Grasses

**DOI:** 10.3390/molecules27072227

**Published:** 2022-03-29

**Authors:** Gabriela Benetel, Thaysa dos Santos Silva, Gisele Maria Fagundes, Katiéli Caroline Welter, Flavia Alves Melo, Annelise A. G. Lobo, James Pierre Muir, Ives C. S. Bueno

**Affiliations:** 1 Department of Animal Science, Universidade de São Paulo–USP, Av. Duque de Caxias Norte, 225, Pirassununga 13635-900, São Paulo, Brazil; gabriela.benetel@usp.br (G.B.); thaysamariana@hotmail.com (T.d.S.S.); katieliwelter@yahoo.com.br (K.C.W.); flaviamelo.zoo@gmail.br (F.A.M.); anneliselobo@usp.br (A.A.G.L.); ivesbueno@usp.br (I.C.S.B.); 2 Department of Animal Science, Universidade Federal de Roraima–UFRR, BR 174, km 12, Boa Vista 69300-000, Roraima, Brazil; 3 Texas A&M AgriLife Research, 1229 North U.S. Hwy 281, Stephenville, TX 76401, USA; jim.muir@ag.tamu.edu

**Keywords:** greenhouse gases, methanogenesis, oregano, ruminants, thyme, NH_3_-N

## Abstract

There is increasing pressure to identify natural feed additives to mitigate methane emissions from livestock systems. Our objective was to investigate the effects of essential oils (EO) extracts star anise (*Illicium verum*), citronella (*Cymbopogon winterianus*), clove bud (*Eugenia caryophyllus*), staigeriana eucalyptus (*Eucalyptus staigeriana*), globulus eucalyptus (*Eucalyptus globulus*), ginger (*Zingiber officinale*), ho wood (*Cinnamomum camphora*), melaleuca (*Melaleuca alternifolia*), oregano (*Origanum vulgare*) and white thyme (*Thymus vulgaris*) on in vitro methane emissions from four rumen-cannulated Nellore cattle grazing a tropical grass pasture as inoculum donors. The semi-automated gas production technique was used to assess total gas production, dry matter degradability, partitioning factor, ammoniacal nitrogen, short-chain fatty acids and methane production. All essential oils were tested in four doses (0, 50, 250 and 500 mg/L) in a randomized block design, arranged with four blocks, 10 treatments, four doses and two replicates. Within our study, oregano and white Thyme EO reduced net methane production at 250 mg/L, without affecting substrate degradation. Essential oils from oregano and white thyme have the potential to modify ruminal fermentation and suppress rumen methanogenesis without negative effects on feed digestibility, indicating promise as alternatives to ionophores for methane reduction in beef cattle.

## 1. Introduction

In 2020, the Brazilian cattle herd was the largest in the world, representing 14.3% of the international herd, with 217 million head [1]. Despite the crisis caused by the new coronavirus pandemic, Brazil was the largest exporter of meat in the world, with 2.2 million tons and 14.4% of the international market [1].

However, despite the recognized importance of livestock to food production and income generation, there is currently much debate about the environmental impact of the activity, mainly related to climate change. Brazilian livestock is singled out for its greenhouse gas (GHG) emissions [2]. Such criticism has been based on low animal performance indexes, verified in animal production systems, based on degraded pastures which are below their production potential [3,4]. Low exploration model efficiency has generated greater amounts of GHG per kg of meat and/or milk produced [3]. In 2019, total emissions from agriculture were 10.7 billion tons of carbon dioxide equivalent, of which methane (CH_4_) from ruminant livestock was the largest contributor (2.8 Gt CO_2_ eq) [5]. This production is directly related to the efficiency of ruminal fermentation and consequently to the loss of energy in the production systems [6]. Brazil has the largest world emissions from agriculture followed by Indonesia and China [5], and only CH_4_ by beef herd contributes to 80% of Brazil emissions [2].

Ruminant contribution to climate change has led to the search for natural alternatives that can be added to grazing cattle diets to mitigate GHG emissions without compromising livestock productivity [7]. Some natural plant products, including essential oils (EO), possess antimicrobial properties capable of manipulating rumen microbiomes [8]. However, data on EO acceptability in cattle feed are scarce. Thus, the objective of our study was to investigate the effects of 10 EO on in vitro rumen methane emissions and degradability in Nelore beef cattle.

## 2. Material and Methods

### 2.1. Essential Oils

Ten essential oils used in this study were commercially obtained from Brazilian industry (Ferquímica^®^, Vargem Grande Paulista, SP, Brazil): star anise (*Illicium verum*), citronella (*Cymbopogon winterianus*), clove bud (*Eugenia caryophyllus*), staigeriana eucalyptus (*Eucalyptus staigeriana*), globulus eucalyptus (*Eucalyptus globulus*), ginger (*Zingiber officinale*), ho wood (*Cinnamomum camphora*), melaleuca (*Melaleuca alternifolia*), oregano (*Origanum vulgare*), and white thyme (*Thymus vulgaris*). The chemical composition of EO was carried out in order to identify the main bioactive compounds according to Wiley Library (Version 8), using a gas chromatograph coupled to a mass spectrometer (GC/MS QP2010 Plus, Shimadzu, Kyoto, Japan), with a diphenyl-dimethyl-polysiloxane capillary column (5% diphenyl and 95% dimethyl polysilozane) with 30 m × 0.25 mm, 0.25 μm, model Rtx^®^-5MS (Bellefonte, PA, USA) at the Multi-User Laboratory for Biochemistry and Instrumental Analysis of the Department of Agribusiness, Food and Nutrition at ESALQ/USP (Piracicaba, Brazil).

### 2.2. Methanogenesis Bioassay

The study was approved by the ethics committee of the Department of Animal Science, College of Animal Science and Food Engineering, University of São Paulo (2416120916).

The in vitro bioassay used a pressure transducer according to methodology proposed by Theodorou et al. (1994) [9] and modified by Mauricio et al. (1999) [10]. Four male Nellore cattle, rumen cannulated, were donors of rumen content. The animals were kept in a tropical grass pasture with free access to water and mineral supplements.

All EO were tested in four dose rates (0, 50, 250 and 500 mg/L) in a randomized block design, arranged with four blocks, 10 treatments (EO), four dose rates and two replicates. The substrate used was a coast-cross Bermudagrass (*Cynodon dactylon*) grass hay, ground in a Willey mill with a 1-mm sieve. Substrates were analyzed (Table 1) for dry matter (DM; ID 930.15), mineral matter (ash; ID 942.05), crude protein (CP; ID 954.01), ether extract (EE; ID 920.39), neutral detergent fiber (NDF; ID 973.18) and acid detergent fiber (ADF; ID 973.18) according to AOAC (1998) [11]. NDF and ADF were determined sequentially with the addition of a-amylase and included residual ash. The collection and preparation of the inoculum followed the recommendations of Bueno et al. (2005) [12]. Glass bottles with a capacity of 160 mL were used; to each bottle, 500 mg of substrate, 25 mL of rumen inoculum and 50 mL of buffered medium were added. After 4, 8, 12, 16, 20, 24 h of incubation, the pressure in the head space was measured. Total gas volume produced in each bottle was estimated according to the equation: V = (6.4278 * P); where V = volume of gases (mL) and P = measured pressure (psi). This equation is accepted for the in vitro experimental conditions for specific methanogenesis bioassays at the Ruminal Fermentability Laboratory. The total gas production after 24 h of fermentation was determined by summing the volume of gases produced in each pressure measurement of the bottles. The pressure inside each flask was measured, and 1.5-mL gas samples were collected in Vacutainer tubes (Becton Dickinson, Franklin Lakes, NJ, USA) for CH_4_ characterization by gas chromatography [13].

After 24 h of incubation, samples of ruminal liquid (2 mL) contained in each bottle were collected for the determination of short-chain fatty acid (SCFA) and NH_3_-N. In vitro DM degradability (IVDMD) and in vitro organic matter degradability (IVOMD) were then determined. Analysis of both were made to correct any differences in mineral content among samples. IVDMD was determined by filtering the fermentation residues in sintered crucibles (porosity n. 1) lined with glass wool and then dried in an oven (105 °C) to determine the residue. Using the difference in residues (ash) of the muffle burning (550 °C), IVOMD was estimated. The partitioning factor (PF), calculated by relating DM degradation to total gas production (mL) in 24 h, was used to compare microbial efficiency (Blummel et al., 1997) [14].

### 2.3. Methane Determination

The CH_4_ quantification was performed by a gas chromatograph (GC-2014, Shimadzu, Kyoto, Japan), equipped with a micro-packed column (ShinCarbon^®^ Restek corporation, Bellefonte, PA, USA) at 60 °C and a dual direct injector and dual FID detector at 120 °C, according to the method described by Santos et al. (2020) [13]. A calibration curve (0, 30, 60 and 90 mL/L) was generated using methane (99% purity) as the standard. The operational conditions were column, injector temperatures were 100 °C and flame ionization detectors were 120 °C.

### 2.4. Short Chain Fatty Acid Determination

An SCFA profile in ruminal liquid was performed by a gas chromatography (GC-2014; Shimadzu, Kyoto, Japan) split-injector, a flame ionization detector and a capillary column (Stabilwax^®^, Restek, Bellefonte, PA, USA) at 145 °C (isothermal), as described by Bueno et al. in 2020 [15]. Acetic, propionic, isobutyric, butyric, isovaleric and valeric acid (99.5% purity, Chem Service, West Chester, PA, USA) were used as quantitative external standards. The operational conditions were: injector and detector temperatures at 250 °C; helium as the carrier gas at 8.01 mL/min; hydrogen flow to the flame jet at 60 kPa; and synthetic air at 40 kPa. The samples were thawed at room temperature and centrifuged at 14,500× *g* for 10 min. The supernatant (800 µL) was transferred to a dry and clean flask with 200 µL formic acid (98–100%) and 100 µL of the internal standard (100 mM 2-ethyl butyric acid, Chem service, West Chester, PA, USA).

### 2.5. NH_3_-N Determination

NH_3_-N concentrations in the ruminal liquid were determined by colorimetry according to the method described by Kulasek (1972) [16] and adapted by Foldager (1977) [17]. In each sample, 10% sodium tungstate was added and then centrifuged at 2000× *g* for 15 min. The supernatant was then transferred to another tube with 1 mL of the salicylic acid buffer and 1 mL of sodium hypochlorite oxidizing solution. Finally, the tubes were placed in water bath (37 °C) until they became green.

### 2.6. Experimental Design and Statistical Analysis

The experimental design was a randomized complete block with two factors (10 essential oils plus four doses) and four blocks (inoculum) with two replications inside each block. Regression analysis performed by the SAS 9.3 program (Institute Inc., Cary, NC, USA) [18] was used for measuring correlations among all investigated parameters.

## 3. Results

### 3.1. Ruminal Degradability

IVDMD, IVOMD and partition factor (PF) of the evaluated EO are shown in Table 2 and Table 3. Among all EO, only white thyme linearly reduced IVDMD.

The PF is a variable that relates degradability and gas production and is expressed by mg of DM (PF_DM_) or OM (PF_OM_) degraded per mL of gas produced during the fermentation process. Oregano EO had a positive linear effect on PF_DM_ and PF_OM_ (Table 3). That means that, with increasing doses of this EO, larger amounts of degraded DM and OM were needed to generate 1 mL of gas. The gas produced represents the partial loss of energy from fermented foods (Mauricio et al., 1999) [10] and, therefore, the increase in the partitioning factor would mean more efficient energy use. White thyme EO showed a quadratic relationship between dose and PF_OM_.

### 3.2. Short-Chain Fatty Acids and N-NH_3_ Quantifications

The NH_3_-N concentrations and short chain fatty acids in the different treatments and levels evaluated are shown in Table 4, Table 5, Table 6, Table 7, Table 8 and Table 9. Citronella and ho wood EO showed a positive linear relationship between NH_3_-N and levels of inclusion. However, the average levels of NH_3_-N collected after 24 h of incubation showed numerically little variation among all EO and tested levels, suggesting a stabilization of the environment and the fermentation process.

Total SCFA concentrations were not affected by any of the analyzed oils. With the exception of the ginger EO that suffered a positive linear effect only for the C_2_ concentration, no other EO changed with concentrations of C_2_, C_3_ and C_4_ (respectively, Table 6, Table 7 and Table 8). However, citronella, globulus, staigeriana, ginger, melaleuca, oregano and white thyme EO induced a positive linear effect for the C_2_:C_3_ ratio (Table 9), thus making the rumen less energetically efficient. The observed effects were dependent on the doses used. The thymol 500 mg/L dose reduced the total concentration of SCFA (−28.5%), the proportion of propionate (−18.4%) and the concentration of NH_3_-N (−31.9%) while increasing the proportion of acetate (C_2_) (+1.8%) and C_2_:C_3_ ratio (+35.5%).

### 3.3. Total Gas Production and Methane Quantifications

Essential oils influenced total gas production (TGP) in the rumen, which had a negative linear relationship (*p* < 0.05) in citronella, ho wood, oregano and white thyme (Table 10). At a dose of 500 mg/L, citronella and ho wood EO decreased TGP by approximately 25%. Oregano and thyme EO caused a sharp decrease in TGP of up to 75%, showing that these substances have high antimicrobial activity in in vitro ruminal conditions.

The production of methane under various EO doses, expressed in net production (net CH_4_) or per gram of degraded dry matter (CH_4_/g DDM), are shown in Table 11. Variations in the amount of CH_4_ can occur even when DM disappearance of the diet does not change with the addition of different EO, with lower CH_4_ production associated with lower TGP.

The same EO that demonstrated negative linear behavior for TGP also showed a drop in the net production of CH_4_, including citronella, ho wood, oregano and white thyme. White thyme and oregano showed a higher potential for mitigating CH_4_ emissions, in doses above 250 mg/L, producing 0.17 and 0.57 mL, respectively, in the highest dose (500 mg/L) against 6.92 mL without the use of any EO.

CH_4_ expressed in mL/g IVDMD represents the relationship between the production of CH_4_ net and IVDMD; the greater the value attributed to this variable implies a greater participation of CH_4_ net production per gram of in vitro degraded dry matter (IVDMD) during the in vitro incubation process. Although the presence of EO changed CH_4_ (mL/g IVDMD) production, variability was such that no statistical relationship was observed between the production of CH_4_ and IVDMD.

## 4. Discussion

Although there is already research on some EO using the in vitro technique of gas production, our work investigated additional EO and variables that were not previously considered. The effects of EO tend to be influenced by their majority variable components (including diet composition), making it difficult to parse out their effect on ruminant nutrition [7]. The possible synergic or antagonistic interactions are difficult to measure and interpret [7].

A reduction of TGP in animals consuming diets with *Lippia turbinate* and *Tagetes minuta* was observed by Garcia et al. (2020) [19]. Those authors observed a reduction in in vitro gas production, without affecting the digestibility of the substrates in intermediate doses. In our study, oregano and white thyme EO caused a drastic reduction in TGP of up to 75%, indicating that these substances have high antimicrobial activity in ruminal conditions. To explain this relationship, Lambert et al. (2001) [20] stated that oregano EO has molecules of thymol and carvacrol, and that these would be most responsible for its antimicrobial effect. These same molecules were observed in the chemical characterization of oregano and thyme EO.

García-García et al. (2011) [21] showed that thymol alone may not be as effective at reducing CH_4_ as the combination between it and carvacrol. When testing the isolated effects of carvacrol or thymol on *Listeria innocua*, they obtained stronger effects for the first compound, while the binary effect of carvacrol plus thymol was more effective in inactivating bacterial growth, evidencing the synergistic effect between the components.

Garcia et al. (2020) [19] observed that increasing doses of EO had progressively inhibitory action in substrates digestibility, with almost total inhibition when using 300 µL/L. In contrast, Khorrami et al.’s (2015) [22] in vivo study observed that thyme and cinnamon essential oils (500 mg/kg DM) did not decrease feed intake and nutrient digestion.

Several factors can influence the TGP and consequently the production of CH_4_ by the animals through rumen fermentation [19]. One of the most important factors in this process is the extent of feed degradation. According to Bueno et al. (2005) [12], the in vitro gas production system estimates the degradation of the feed (substrate). Previous studies also suggest that in vitro gas production assays should be complemented with residue determination at the end of the incubation process, since the gas measurements alone provide an incomplete explanation. The determination of the final residue indicates the amount of substrate that was effectively used during the in vitro fermentation process (Getachew et al., 1998) [23].

Castilejos et al. (2006) [24] and Fraser et al. (2007) [25], when testing 500 ppm thymol (the primary compound of thyme EO) and cinnamaldehyde, also observed a reduction in the disappearance of IVDMD with these substances, as we also found. Other studies using EO showed a reduction in feed degradation [26,27], which would be a disadvantage for animal production in the use of this additive.

The search for EO that reduce ruminal CH_4_ emissions without affecting feed degradation is important to improve feed efficiency and thus contribute to innovation in green technologies [19]. Feeds with low degradation may make it difficult for rumen microorganisms to extract substrates and, consequently, result in nutrient limitation [28].

In our study, some EO, such as citronella, ho wood, oregano and thyme, likely induced hydrophobic characteristics on the soluble portions of the feed substrate, preventing microbial attack and its metabolism. This was evidenced when analyzing the TGP drop and the maintenance in the degradability of the substrate with increasing inclusion levels.

The partition factors for both DM and OM in oregano and white thyme EO in our research were superior to the other EO when evaluated at a dosage of 500 mg/L. This may be explained by the low production of gases with a concurrent lower substrate disappearance in the presence of white thyme EO and constant substrate disappearance when oregano was added.

The quantification of ammonia concentration is an important indication of nitrogen use efficiency during in vitro bioassays, since 60% to 80% of the nitrogen incorporated by rumen microorganisms comes from ammonia [29]. Its concentration may be affected both by the degradability content of CP present in the substrate and by rumen microorganism use. Feed CP functions as a source of rumen NH_3_-N for ruminal microorganisms so it is essential to have sufficient energy available. Thus, rumen NH_3_-N is a direct result of the quantity generated and the quantity used by rumen microorganisms [30].

All the EO evaluated presented little variation in NH_3_-N content; however, the EO of citronella and ho wood had a positive linear relation between NH_3_-N and inclusion levels, suggesting a stabilization of the microbiota environment. The concentrations were always sufficient to support microbiota growth, all being well above the minimum value reported by Satter and Slyter (1974) [31], of 5 mL/dL. In addition, they were above 13 mg/dL—the minimum value necessary to avoid compromising the availability of N for microorganisms, and the ingestion and digestibility of fiber [28].

Benchaar et al. (2007) [32] did not observe an effect of cinnamon (400 mg/L), oregano (200 mg/L) and thymol (200 mg/L) EO on rumen NH_3_-N concentration in relation to the treatment without additives. Cardozo et al. (2005) [33], when testing 0.3 mg/L of garlic, cinnamon and oregano EO, also did not observe a difference compared to the control diets without EO. However, garlic EO doses of 30 and 300 mg/L reduced ammonia concentration by 32.7% and 55.4%, for cinnamon EO by 31.6% and 61.9%, and for oregano EO by 26.9% and 64.5%, respectively. According to those authors, either EO decreased the deamination or microorganisms used the peptides and amino acids as a source of nitrogen, with a consequent reduction in NH_3_-N.

More important than analyzing the effect of each EO on each variable related to ruminal fermentation is to analyze the influence and consequences that each exerts on the other [34]. The absence of effects on total concentrations of SCFA can also be seen as a positive if it is accompanied by increased feed degradability as well as decreases in C_2_:C_3_ ratios and the production of CH_4_.

A secondary metabolite can have a beneficial effect against methane-producing microorganisms but can also reduce the total concentration of SCFA and/or the degradability of feed in ruminants [35]. There is an inverse relationship between C_3_ and CH_4_ production; the metabolic route of C_3_ production, besides serving as a drain of H^+^, still generates less of this ion compared to the C_2_ or C_4_ route [28,36]. By increasing the C_3_ production, there is more competition with methanogenic archaea for substrate, generating less CH_4_ production.

Citronella, globulus, staigeriana, ginger, melaleuca, oregano and white thyme OE had a positive linear relationship with C_2_:C_3_ ratios, indicating that the rumen was less energy efficient. However, the C_2_:C_3_ ratio and the maintenance of the degradability of coast-cross hay suggests that the addition of EO led to a decline in selectivity in cellulolytic bacteria, responsible for the degradation of fibrous substrates and the main producers of C_2_.

Diets rich in concentrate are more prone to low ruminal pH, enhancing the effects of EO. According to Cardozo et al. (2005) [33], cinnamon EO and its main component, cinnamaldehyde, increase C_2_:C_3_ ratios in a rumen incubation medium with pH 7.0 while in a medium with pH 5.5, it causes a reduction in the C_2_:C_3_ ratio. Calsamiglia et al. (2007) [37] also found that thymol EO was more effective at pH 5.5 than at 6.5.

Castillejos et al. (2006) [24] in a 24 h in vitro assay using a substrate with 60% forage evaluated eugenol, guaiacol, limonene, thymol and vanillin EO at doses of 5, 50, 500 and 5000 mg/L. The effects observed by these authors depended on the doses used. The dose of 500 mg/L of thymol EO reduced the total concentration of SCFA (−28.5%), the proportion of propionate (−18.4%) and the concentration of NH_3_-N (−31.9%) while there was an increase in the proportion of C_2_ (+1.8%) and in the C_2_:C_3_ ratio (+35.5%).

In an experiment testing thyme, oregano and cinnamon EO, and their respective pure compounds—thymol, carvacrol and cinnamaldehyde—on rumen activity, Macheboeuf et al. (2008) [38] observed results similar to those found in our experiment; however, cinnamon EO and its main compound cinnamaldehyde in concentrations lower than 3 mmol/L did not affect the production of CH_4_ at 5 mmol/L. There was no production of CH_4_ due to hydrogen accumulation in the headspace of the vial. Oregano, thyme, thymol and carvacrol EO were more toxic to ruminal microbiota, due to the lower dose needed (1, 1.5, 2.0 and 2.5 mmol/L, respectively) to inhibit the ruminal production of CH_4_. This result reinforces the theory previously discussed that EO effects on ruminal microbiota depend on the dose used.

The variable CH_4_, expressed in mL/g IVDMD, represents the relationship between the production of net CH_4_ and IVDMD. The greater the value attributed to this variable, the greater the participation of net CH_4_ produced per gram of degraded dry matter (IVDMD) during the in vitro incubation process. Although the EO in our trial changed CH_4_ (mL/g IVDMD), there were no relationships observed between the production of CH_4_ and IVDMD. This can be attributed to the high standard error of the mean, since CH_4_, expressed as mL/g IVDMD, associates variability in gas measurements, CH_4_ liquid concentration, and residual non-degradable DM.

## 5. Conclusions

Our study showed that oregano and white thyme EO had the greatest reduction on CH_4_ production compared with other EO in vitro when using rumen liquid from cattle consuming high-fiber grass diets. However, these EO did not affect substrate disappearance and provided the lowest net CH_4_ production. Taken together, these preliminary data lead us to believe that these two EO effectively suppress rumen CH_4_ emissions without negatively affecting digestibility. However, additional research, especially in vivo, is needed with these two EO looking at a greater variety of diets and substrates with sufficient replication to reduce standard errors. We further recommend testing these EO in in vivo trials.

## Figures and Tables

**Table 1:** Chemical composition of substrate used in the in vitro bioassay (g/kg DM).

Substrate	DM	OM	MM	CP	NDF	ADF
Coast cross hay	880	927	73	165	743	42.1

ADF, acid detergent fiber; DM, dry matter; CP, crude protein; MM, mineral matter; OM, organic matter; NDF, neutral detergent fiber.

**Table 2:** Effects of essential oils on the in vitro dry matter (IVDMD) and organic matter (IVOMD) degradabilities after 24 h of incubation.

Essential Oils	Doses (mL/g)				M	EPM	*p*-Value	
	0	50	250	500			L	Q
**IVDMD (% of Herbage DM)**								
Star anise	24.67	28.76	21.05	20.08	23.64	4.31	0.192	0.411
Citronella	24.67	28.07	21.17	31.86	26.44	5.10	0.441	0.680
Clove bud	24.67	33.20	25.11	21.17	26.04	5.81	0.276	0.561
Globulus	24.67	28.47	27.79	26.09	26.76	4.74	0.849	0.958
Staigeriana	24.67	28.22	24.23	23.19	25.08	5.27	0.551	0.840
Ginger	24.67	33.92	26.09	24.64	27.33	4.61	0.365	0.674
Ho wood	24.67	27.48	26.99	27.68	26.71	4.58	0.851	0.792
Melaleuca	24.67	28.44	31.25	23.29	26.91	5.93	0.864	0.882
Oregano	24.67	25.45	25.47	27.07	25.66	5.52	0.438	0.736
White thyme	24.67	36.28	21.17	18.54	25.16	4.78	0.041 ^1^	0.136
**IVDOM (% of Herbage DM)**								
Star anise	25.82	27.60	21.29	19.04	23.44	3.24	0.074	0.202
Citronella	25.82	27.66	21.20	19.28	23.49	3.71	0.130	0.312
Clove bud	25.82	31.71	25.04	20.73	25.82	4.81	0.203	0.453
Globulus	25.82	28.35	27.73	24.02	26.48	3.78	0.524	0.746
Staigeriana	25.82	28.99	24.18	23.41	25.60	4.12	0.411	0.712
Ginger	25.82	33.01	26.84	25.01	27.67	3.47	0.298	0.588
Ho wood	25.82	28.33	31.04	24.39	27.40	3.33	0.574	0.487
Melaleuca	25.82	28.83	31.56	24.98	27.80	4.74	0.790	0.830
Oregano	25.82	26.30	26.34	22.71	25.29	4.27	0.162	0.324
White thyme	25.82	34.58	21.05	20.62	25.52	3.72	0.099	0.229

L = linear effect; M = means; Q = Quadratic effect; SEM = Standard error of the mean. * Equation: ^1^ y = 32.24 − 0.037x.

**Table 3:** Effects of essential oils on the dry matter partition factor (PF_DM_) and organic matter (PF_OM_) after 24 h of in vitro incubation.

Essential Oils	Doses (mL/g)				M	SEM	*p*-Value	
	0	50	250	500			L	Q
**PF_DM_ (mL DMS/g Produced Gases)**								
Star anise	2.62	3.06	2.13	2.84	2.66	0.71	0.870	0.774
Citronella	2.62	2.89	2.04	3.44	2.75	0.79	0.518	0.497
Clove bud	2.62	4.07	2.59	2.77	3.01	0.89	0.555	0.830
Globulus	2.62	2.78	2.26	2.79	2.61	0.60	0.990	0.965
Staigeriana	2.62	2.93	2.19	2.25	2.50	0.64	0.460	0.718
Ginger	2.62	3.19	2.05	2.13	2.50	0.54	0.232	0.430
Ho wood	2.62	2.68	2.43	4.18	2.98	0.72	0.166	0.342
Melaleuca	2.62	2.46	2.45	1.99	2.38	0.50	0.783	0.946
Oregano	2.62	2.28	3.29	13.2	5.35	0.99	0.014 ^1^	0.025
White thyme	2.62	3.42	2.10	7.58	3.93	1.04	0.364	0.278
**PF_OM_ (mL OMD/g Produced Gases)**								
Star anise	2.73	2.91	2.15	2.64	2.61	0.57	0.733	0.730
Citronella	2.73	2.83	2.04	2.99	2.65	0.60	0.721	0.554
Clove bud	2.73	3.85	2.57	2.73	2.97	0.78	0.547	0.822
Globulus	2.73	2.76	2.54	2.58	2.65	0.50	0.762	0.941
Staigeriana	2.73	2.98	2.16	2.25	2.53	0.52	0.347	0.578
Ginger	2.73	3.08	2.09	2.14	2.51	0.43	0.167	0.325
Ho wood	2.73	2.76	2.81	3.65	2.99	0.55	0.263	0.491
Melaleuca	2.73	2.51	2.49	2.15	2.47	0.43	0.840	0.953
Oregano	2.73	2.35	3.20	11.21	4.87	0.79	0.010 ^2^	0.016
White thyme	2.73	3.26	2.09	7.97	4.01	1.00	0.093	0.038 ^3^

L = Linear effect; M = Means; Q = Quadratic effect; SEM = Standard error of the mean. * Equations: ^1^ y = 2.21 + 0.008x; ^2^ y = 2.21 + 0.008x; ^3^ y = 3.36 − 0.017x + 0.00005x^2^.

**Table 4:** Effects of essential oils on the concentration of ammonia nitrogen (NH_3_-N, in mg/dL) after 24 h of in vitro incubation.

Essential Oils	Doses (mg/dL)				M	SEM	*p*-Value	
	0	50	250	500			L	Q
Star anise	17.08	15.59	16.44	16.67	16.45	0.63	0.439	0.747
Citronella	17.08	16.00	17.92	18.99	17.50	0.72	0.010 ^1^	0.041
Clove bud	17.08	16.19	17.14	17.27	16.92	0.60	0.232	0.476
Globulus	17.08	16.10	14.72	14.51	15.60	1.46	0.355	0.616
Staigeriana	17.08	15.67	14.91	17.41	16.27	0.86	0.391	0.192
Ginger	17.08	17.72	16.37	14.08	16.31	1.17	0.083	0.192
Ho wood	17.08	15.93	16.32	18.77	17.03	0.64	0.009 ^2^	0.039
Melaleuca	17.08	14.68	15.55	16.06	15.84	1.48	0.911	0.960
Oregano	17.08	16.80	16.60	16.33	16.70	1.02	0.698	0.903
White thyme	17.08	15.22	15.57	15.81	15.92	1.10	0.847	0.857

L = Linear effect; M = Means; Q = Quadratic effect; SEM = Standard error of the mean. * Equations: ^1^ y = 16.10 + 0.006x; ^2^ y = 15.47 + 0.0005x.

**Table 5:** Effects of essential oils on the total concentration of short chain fatty acids (SCFA, in mM) after 24 h of in vitro incubation.

Essential Oils	Doses (mL/g)				M	SEM	*p*-Value	
	0	50	250	500			L	Q
Star anise	11.52	11.59	20.66	12.44	14.05	4.49	0.767	0.377
Citronella	11.52	9.60	24.66	12.80	14.64	5.60	0.698	0.250
Clove bud	11.52	14.34	26.95	14.76	16.89	5.61	0.690	0.221
Globulus E.	11.52	15.98	13.02	19.35	14.97	4.07	0.357	0.598
Staigeriana E.	11.52	13.41	19.38	18.79	15.77	3.43	0.170	0.274
Ginger	11.52	14.35	15.96	24.60	16.61	4.06	0.055	0.159
Ho wood	11.52	16.69	18.98	17.32	16.13	3.59	0.494	0.702
Melaleuca	11.52	13.58	16.05	26.22	16.84	3.78	0.297	0.582
Oregano	11.52	11.66	16.00	3.99	10.79	3.91	0.437	0.291
White thyme	11.52	10.68	16.28	17.57	14.01	7.06	0.229	0.488

L = Linear effect; M = Means; Q = Quadratic effect; SEM = Standard error of the mean.

**Table 6:** Effects of essential oils on acetic acid (mM) production after 24 h of in vitro incubation.

Essential Oils	Doses (mL/g)				M	SEM	*p*-Value	
	0	50	250	500			L	Q
Star anise	7.54	7.90	14.92	8.58	9.74	3.35	0.734	0.337
Citronella	7.54	6.62	18.61	9.54	10.58	4.29	0.410	0.211
Clove bud	7.54	10.36	20.21	9.49	11.90	4.13	0.775	0.155
Globulus	7.54	10.50	9.32	14.09	10.36	2.93	0.240	0.476
Staigeriana	7.54	9.48	14.72	14.13	11.47	2.55	0.111	0.166
Ginger	7.54	10.32	11.75	18.07	11.92	3.01	0.043 ^1^	0.134
Ho wood	7.54	11.49	14.08	11.28	11.10	2.60	0.587	0.639
Melaleuca	7.54	9.83	11.93	19.52	12.21	2.88	0.239	0.490
Oregano	7.54	7.98	10.77	2.27	7.14	2.56	0.376	0.222
White thyme	7.54	7.60	11.98	12.69	9.95	5.18	0.209	0.466

L = Linear effect; M = Means; Q = Quadratic effect; SEM = Standard error of the mean. * Equation: ^1^ y = 8.19 + 0.019x.

**Table 7:** Effects of essential oils on propionic acid (mM) production after 24 h of in vitro incubation.

Essential Oils	Doses (mL/g)				M	SEM	*p*-Value	
	0	50	250	500			L	Q
Star anise	2.42	2.17	3.34	1.97	2.48	0.69	0.856	0.487
Citronella	2.42	1.71	3.45	1.37	2.24	0.82	0.617	0.325
Clove bud	2.42	2.46	4.11	2.47	2.87	0.94	0.155	0.445
Globulus	2.42	3.17	2.09	2.99	2.67	0.70	0.918	0.793
Staigeriana	2.42	2.36	2.66	2.59	2.51	0.55	0.766	0.939
Ginger	2.42	2.35	2.50	3.85	2.78	0.66	0.143	0.281
Ho wood	2.42	3.08	2.88	3.62	3.00	0.65	0.346	0.643
Melaleuca	2.42	2.31	2.35	3.46	2.64	0.57	0.873	0.977
Oregano	2.42	2.20	2.46	0.43	1.88	0.68	0.149	0.220
White thyme	2.42	1.84	2.24	2.05	2.14	0.92	0.605	0.824

L = Linear effect; M = Means; Q = Quadratic effect; SEM = Standard error of the mean.

**Table 8:** Effects of essential oils on butyric acid (mM) production after 24 h of in vitro incubation.

Essential Oils	Doses (mL/g)				M	SEM	*p*-Value	
	0	50	250	500			L	Q
Star anise	1.14	1.14	1.80	1.51	1.40	0.39	0.445	0.513
Citronella	1.14	0.96	2.01	1.45	1.39	0.44	0.473	0.379
Clove bud	1.14	1.15	1.85	1.91	1.51	0.42	0.177	0.355
Globulus	1.14	1.37	1.23	1.77	1.38	0.36	0.335	0.572
Staigeriana	1.14	1.19	1.51	1.63	1.37	0.31	0.265	0.525
Ginger	1.14	1.28	1.30	2.04	1.44	0.35	0.114	0.248
Ho wood	1.14	1.57	1.58	2.05	1.59	0.37	0.195	0.419
Melaleuca	1.14	1.11	1.37	2.65	1.57	0.35	0.269	0.554
Oregano	1.14	1.12	2.10	1.23	1.40	0.66	0.501	0.540
White thyme	1.14	0.93	1.52	2.68	1.57	0.93	0.117	0.235

L = Linear effect; M = Means; Q = Quadratic effect; SEM = Standard error of the mean.

**Table 9:** Effects of essential oils on the ratio of acetic and propionic acids after 24 h of in vitro incubation.

Essential Oils	Doses (mL/g)				M	SEM	p-Value	
	0	50	250	500			L	Q
Star anise	3.09	3.49	4.21	4.40	3.80	0.47	0.377	0.094
Citronella	3.09	3.62	5.23	6.76	4.67	0.51	0.001 ^1^	0.001
Clove bud	3.09	4.08	4.83	3.60	3.90	0.50	0.628	0.079
Globulus	3.09	3.28	4.60	4.58	3.89	0.46	0.022 ^2^	0.027
Staigeriana	3.09	4.10	5.32	5.44	4.49	0.46	0.008 ^3^	0.006
Ginger	3.09	4.09	4.82	4.65	4.16	0.42	0.049 ^4^	0.029
Ho wood	3.09	3.75	4.87	3.27	3.74	0.51	0.805	0.205
Melaleuca	3.09	4.24	4.93	5.43	4.42	0.59	0.047 ^5^	0.080
Oregano	3.09	4.76	4.41	4.79	4.26	0.48	0.052 ^6^	0.112
White thyme	3.09	3.26	4.86	5.03	4.06	0.59	0.058 ^7^	0.113

L = Linear effect; M = Means; Q = Quadratic effect; SEM = Standard error of the mean. * Equations: ^1^ y = 3.10 + 0.008x; ^2^ y = 3.07 + 0.004x; ^3^ y = 3.50 + 0.005x; ^4^ y = 3.52 + 0.003x; ^5^ y = 3.51 + 0.004x; ^6^ y = 3.15 + 0.004x; ^7^ y = 2.89 + 0.005x.

**Table 10:** Effects of essential oils on the production of total gases (mL/g DM) after 24 h of in vitro incubation.

Essential Oils	Doses (mL/g)				M	SEM	*p*-Value	
	0	50	250	500			L	Q
Star anise	96.65	101.19	102.05	81.66	95.39	7.95	0.137	0.204
Citronella	96.65	102.41	103.87	73.11	94.01	6.37	0.008 ^1^	0.006
Clove bud	96.65	88.06	98.55	82.87	91.53	7.20	0.342	0.475
Globulus	96.65	104.99	110.60	99.63	102.97	7.42	0.890	0.571
Staigeriana	96.65	101.44	116.40	107.23	105.43	6.21	0.370	0.230
Ginger	96.65	111.48	134.76	120.39	115.82	10.21	0.266	0.139
Ho wood	96.65	104.36	111.84	72.86	96.43	6.46	0.026 ^2^	0.008
Melaleuca	96.65	119.49	129.24	118.27	115.91	9.64	0.549	0.272
Oregano	96.65	114.39	87.00	23.93	80.49	10.32	0.001 ^3^	0.002
White thyme	96.65	108.92	104.20	26.55	84.08	8.60	0.009 ^4^	0.001

L = linear effect; M = means; Q = Quadratic effect; SEM = Standard error of the mean; TGP = Total gas production. * Equations: ^1^ y = 110.49 − 0.064x; ^2^ y = 107.56 − 0.057x; ^3^ y = 112.70 − 0.151x; ^4^ y = 114.65 − 0.128x.

**Table 11:** Effects of essential oils on methane production after 24 h of in vitro incubation.

Essential Oils	Doses (mL/g)				M	SEM	*p*-Value	
	0	50	250	500			L	Q
**Net CH_4_ (%)**								
Star anise	6.92	8.13	9.01	7.52	7.90	0.64	0.699	0.344
Citronella	6.92	7.96	10.3	7.26	8.11	0.42	0.584	0.001 ^1^
Clove bud	6.92	7.90	9.50	6.40	7.68	0.78	0.332	0.082
Globulus	6.92	8.55	9.87	9.57	8.73	0.62	0.120	0.141
Staigeriana	6.92	8.45	10.93	11.43	9.43	0.45	0.001 ^2^	0.001
Ginger	6.92	8.16	10.79	10.89	9.19	0.45	0.001 ^3^	0.001
Ho wood	6.92	8.05	9.42	5.18	7.39	0.59	0.046 ^4^	0.003
Melaleuca	6.92	9.13	11.30	9.95	9.32	0.50	0.171	0.006 ^5^
Oregano	6.92	9.23	6.63	0.17	5.74	0.93	0.001 ^6^	0.001
White thyme	6.92	9.05	8.53	0.57	6.27	0.48	0.003 ^7^	<0.0001
**CH_4_ (mL/g IVDMD)**								
Star anise	67.81	81.04	177.24	102.40	107.12	52.52	0.645	0.440
Citronella	67.81	69.39	125.12	117.62	94.99	35.16	0.350	0.542
Clove bud	67.81	65.73	113.40	92.28	84.81	25.39	0.471	0.540
Globulus	67.81	84.53	85.02	101.79	84.79	19.11	0.418	0.722
Staigeriana	67.81	79.98	129.02	131.32	102.03	31.04	0.196	0.373
Ginger	67.81	54.12	130.35	109.89	90.54	30.75	0.250	0.350
Ho wood	67.81	66.03	128.45	72.47	83.69	23.75	0.957	0.995
Melaleuca	67.81	107.53	106.01	82.60	90.99	33.20	0.925	0.855
Oregano	67.81	86.84	131.71	3.92	72.57	39.70	0.426	0.184
White thyme	67.81	57.19	118.88	35.75	69.91	31.47	0.873	0.388

L = Linear effect; M = Means; Q = Quadratic effect; SEM = Standard error of the mean. * Equations: ^1^ y = 7.63 + 0.021x − 0.00004x^2^; ^2^ y = 8.32 + 0.007x; ^3^ y = 8.18 + 0.006x; ^4^ y = 8.66 − 0.005x; ^5^ y = 7.98 + 0.024x − 0.00004x^2^; ^6^ y = 9.31 − 0.016x; ^7^ y = 9.53 − 0.013x.

## Data Availability

Not applicable.
